# COVID-19 SOCIAL ISOLATION IN BRAZIL: EFFECTS ON THE PHYSICAL ACTIVITY ROUTINE OF FAMILIES WITH CHILDREN

**DOI:** 10.1590/1984-0462/2021/39/2020159

**Published:** 2020-11-11

**Authors:** Cristina dos Santos Cardoso de Sá, André Pombo, Carlos Luz, Luis Paulo Rodrigues, Rita Cordovil

**Affiliations:** a Universidade Federal de São Paulo, Santos, SP, Brazil.; b Faculdade de Motridicade Humana, Universidade de Lisboa, Lisbon, Portugal.; c Escola Superior de Educação, Instituto Politécnico de Lisboa, Lisbon, Portugal.; d Escola Superior Desporto e Lazer de Melgaço, Instituto Politécnico de Viana do Castelo, Viana do Castelo, Portugal.; e Centro de Investigação em Desporto, Saúde e Desenvolvimento Humano, Vila Real, Portugal.; f CIPER, Universidade de Lisboa, Portugal.

**Keywords:** Quarantine, Screen time, Sedentary behavior, Motor activity, Child development, Quarentena, Tempo de tela, Comportamento sedentário, Atividade motora, Desenvolvimento infantil

## Abstract

**Objective:**

To identify how Brazilian families with children aged under 13 years face the period of social isolation resulting from the COVID-19 pandemic, especially regarding the time spent on physical activity (PA), intellectual activity, games, outdoor activities and screen.

**Methods:**

An anonymous online survey was launched on March 24, 2020 in Brazil to assess how families with children aged up to 12 years are adjusting their daily routines to this situation. In the survey, each family reported the daily time each child spent in sedentary activity (sum of intellectual activities, play time on screen, playing without PA) and PA (sum of playing with PA and PA).

**Results:**

The main findings based on data from 816 children indicate that most parents consider there was a reduction in the time that children spend practicing PA; increase in screen play time and family activities, differences between sex were found regarding screen play time (boys>girls) and in playing without PA (girls>boys), and there was an age effect for all categories analyzed, with a tendency to increase the total time of sedentary lifestyle and complementary reducing the time of PA over age.

**Conclusions:**

The household routines of families during the period of social isolation resulting from the COVID-19 pandemic confirm the general reduction tendency in PA time during childhood.

## RESUMO

**Objetivo:**

Identificar como as famílias brasileiras com crianças abaixo de 13 anos enfrentam o período de distanciamento social decorrente da pandemia de COVID-19, principalmente no que diz respeito ao tempo gasto em atividade física (AF), atividade intelectual, brincadeiras, atividades ao ar livre e em tela.

**Métodos:**

Uma pesquisa on-line anônima foi lançada em 24 de março de 2020 no Brasil para avaliar como as famílias com crianças de até 12 anos estão ajustando suas rotinas diárias a essa situação. Na pesquisa, cada família relatou o tempo diário em que cada criança esteve envolvida em atividade sedentária (somatório de atividades intelectuais, tempo lúdico de tela, brincar sem AF) e AF (somatório do brincar com AF e da AF).

**Resultados:**

Os principais achados com base em dados de 816 crianças indicam que a maioria dos pais considera que houve redução no tempo em que as crianças passam praticando AF e aumento do tempo lúdico de tela e das atividades em família. Diferenças entre o sexo foram encontradas no tempo lúdico de tela (meninos>meninas) e no brincar sem AF (meninas>meninos), bem como efeito da idade para todas as categorias analisadas, com tendência para aumento do tempo total de sedentarismo e diminuição complementar do tempo de AF ao longo da idade.

**Conclusões:**

As rotinas domiciliares das famílias durante o período de distanciamento social decorrente da pandemia de COVID-19 confirmam a tendência geral decrescente do tempo de AF na infância.

## INTRODUCTION

In December, 2019, a series of inexplicable cases of pneumonia was reported in Wuhan, China.^1^ In January, 2020, the World Health Organization (WHO) classified this epidemic as a public health emergency of international concern^2^, and, in February, as the Coronavirus 2019 disease (COVID-19), which was named as Severe Acute Respiratory Syndrome coronavirus-2 (SARS-CoV-2) by the coronavirus study group of the International Committee on Taxonomy of Viruses.^3^

In the beginning of April, there were 1,500,830 confirmed cases and 87,706 deaths around the world.^4^ On the same date, Brazil accounted for 15,927 confirmed cases and 800 dead by the new coronavirus.^5^ With the advance of the transmission in several countries, and the occurrence of community transmission, social containment measures have been proposed around the world, including Brazil. The WHO, in the absence of efficient treatments, recommends isolation of suspected cases and social isolation, essential strategies to contain the exponential growth of cases and the overload in health services.^6^

With these measures, the school system has been shut down in several countries, as well as non-essential public and private services; companies have shifted their employees to the home-office system, and millions of families were asked to stay at home.

In Brazil, despite the presence of differences regarding social *isolation* in the five regions of the country, face-to-face school activity was 100% suspended since the second week of March.^7^ Therefore, the children are staying at home, starting what apparently will be a long period of movement restriction, without any organized physical activity (PA) or possibility to play outdoors, thus making children more prone to harmful behavior, such as excessive sedentary behaviors.^8^

We have never experienced a situation like that, in which millions of children all around the world are confined in their households, and separated from their peers, for a long period of time. Therefore, we do not know how these children and their families will act during this period of time, nor which adaptations will be made, as well as options as possibilities to use the time in confinement.

Therefore, identifying the household routines of children in social isolation to understand the behavior of families, understanding how the motor variables change and adjust every day and, afterwards, intervening with specific strategies, are essential actions to minimize the negative effects of a prolonged period of confinement.

This study aims at verifying how Brazilian families with children aged less than 13 years face this problematic period, regarding time spent on PA, intellectual activity, games, outdoor activities and screen time.

## METHOD

This is a cross-sectional, descriptive study, which is part of an international analysis hosted by Universidade de Lisboa (UL), to understand the behavior of children aged less than 13 years during the period of confinement resulting from the COVID-19 pandemic.

To assess how families with children aged from zero to 12 years are dealing with the confinement caused by COVID-19, we created a questionnaire based on LimeSurvey, free software to apply online questionnaires that can use databases for data persistence, housed in Faculdade de Motricidade Humana at UL. This questionnaire was elaborated by a committee of experts in the field, and tested in 15 families (pre-test). After adjustments in the presentation of the responses regarding the number of hours of activities performed by children, it was publicized.

The study was approved by the Research Ethics Committee of Universidade Federal de São Paulo (UNIFESP) (Certificate of Ethical Appreciation] 30930120.2.000.5505 n. 0413/2020). In Brazil, the questionnaire was launched online on March 24, and publicized in the social media (Facebook, Instagram, WhatsApp) and by e-mail, according to the snowball technique. The questionnaire is anonymous, takes five minutes to be filled out and includes four sections:

- Family: family composition, number of children and adults who are at home, and how many are practicing their professional activity or working from the household.
- Household characteristics: type and characteristics of the house, existence or not of an indoor and an outdoor space for PA.
- Household routines: level of concern regarding the COVID-19 situation and way in which family routines are being adjusted (time of PA, screen time, sleep, family activities).
- Children’s routine: characterization of each child (age, sex, health status) and hours spent on different activities on the previous day.

The questionnaires answered by the parents/tutors of all children aged less than 13 years in the same household, during the period of social isolation, from March 25 to April 24, 2020, were included in this study, reaching 1,352 responses. All participants read the information about the investigation and agreed with the conditions by clicking to continue on the first page of the poll. Participants can give up at any time, by not continuing with the questionnaire or not sending the information. After cleaning the database, the responses related to 816 children aged from zero to 12 years (410 boys and 403 girls, and three with no identification) were included here; the answers regarding 536 children (39.6% of the children reported initially) were excluded because of missing or wrong information (for instance, more than 24 hours reported in one day, or no time of sleep reported for children).

The children were divided in four age groups:

- G1: 0-2 years old (n=187).
- G2: 3-5 years old (n=206).
- G3: 6-9 years old (n=285).
- G4: 10-12 years old (n=138).

Descriptive statistics and frequency analysis were used to describe the environment and the routines of the families and children during this period. Five activity categories were analyzed:

- Intellectual activity: school activities and online classes.
- Playful screen time: games, movies, social media, internet, audio and video calls.
- Playing without PA: reading, drawing, painting, board games, cards, Lego etc.
- Playing with PA: hide and seek, run and catch, running, jumping rope etc.
- PA: organized PA in closed environments, outdoor PA, walking the dog.

The three first categories (intellectual activity, playful screen time and playing without PA) were included to calculate the general sedentary time, and the two last categories (playing with PA and PA), to calculate the general PA time. Separated two-way ANOVA tests (age group per gender) were performed to investigate how the different activities and routines of children who are confined, and their families, are being organized according to age and sex of the children.

## RESULTS

Most of the children live in an apartment (56%), and do not have a place dedicated to physical exercises (86.6%), like gym or gymnastics room, in their households. Regarding the outdoors: 27.7% does not have daily access to an outdoor area; 54.4% has an external area of up to 12m^2^; and 17.9% has access to an area bigger than 12 m^2^. It is worth to mention that 52.9% of the families reported it was not easy to maintain social isolation among the children, and 27.7% reports otherwise.

**Figure 1:**
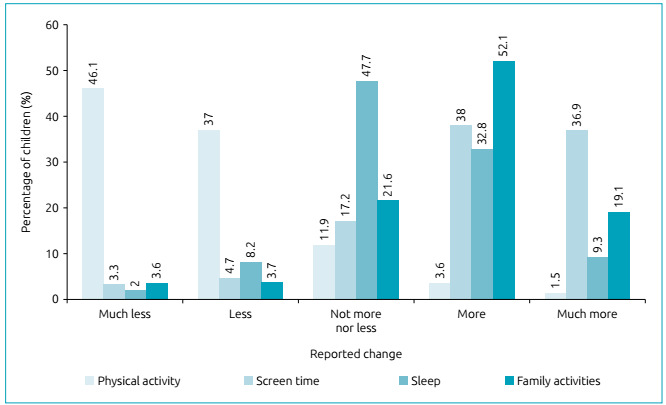
Changes in the time the children spent performing different activities during social isolation, when compared to the previous school time (information reported by the parents).

Before isolation, 67.8% of the children practiced PA at least twice a week. Figure 1 shows changes in the family routine regarding the organization of time during social isolation. Most parents pointed to reduction in the levels of PA among their children: 46.1% reports that children are doing much less PA, and 37% says that PA is less frequent than that performed during the school period. Screen time, sleep and family activities increased. Most parents state that screen time increased: 38% reports it is higher than in regular school hours, and 36.9% says it is much higher. There was an increase in the performance of family activities: 52.1% claims to be having more family activities than before isolation, and 19.1% reports that these activities are more frequent (Figure 1).

**Figure 2:**
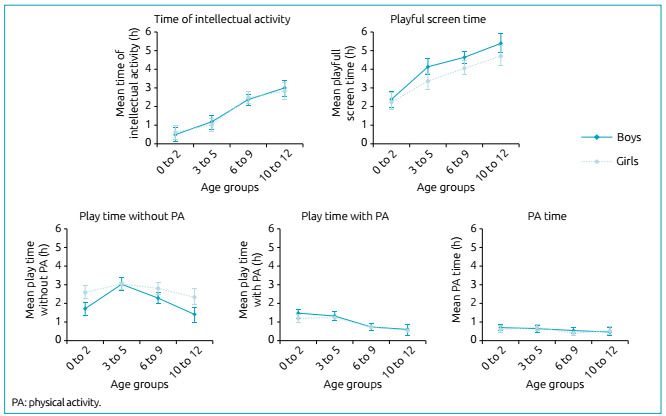
Mean time (hours) of children, as reported by their parents, spent on different activities during social isolation, according to age group and sex. The error bar represents the 95% confidence.

The results referring to the effect of age and sex on the time spent by the children in the different groups of activities carried out during the day are demonstrated in Table 1 and Figure 2. The intellectual activity increases with the age groups (Figure 2). We observed there is no difference between genders, but the effect of age is significant (Table 1), being this activity less frequent in G1 (p<0.001) and p=0.014 between G3 and G4.

**Table 1:** Mean, standard deviation, and results of the variance analysis on the effect of age, sex and their interaction in the groups of activities carried out by the children during the day, as reported by their parents.

	Group	Sex	Mean±SD	Two-way ANOVA
Time of intellectual activity (hours)	0 to 2 years old	M	0.5±0.9	Age: F_3,805_=63.279; p<0.001 Sex: F_1,805_=0.023; p=0.881 Age*sex: F_3.805_=0.306; p=0.821
		F	0.6±1.6	
	3 to 5 years old	M	1.2±1.5	
		F	1.0±1.1	
	6 to 9 years old	M	2.4±2.0	
		F	2.5±2.1	
	10 to 12 years old	M	3.0±2.6	
		F	2.8±2.5	
Playful Screen Time (hours)	0 to 2 years old	M	2.4±1.9	Age: F_3,805_=48.850; p<0.001 Sex: F_1,805_=10.936; p=0.001 Age*sex: F_3,805_=0.790; p=0.500
		F	2.3±2.2	
	3 to 5 years old	M	4.2±2.2	
		F	3.4±2.0	
	6 to 9 years old	M	4.6±2.4	
		F	4.0±2.1	
	10 to 12 years old	M	5.4±2.3	
		F	4.7±2.0	
Playing time without physical activity (hours)	0 to 2 years old	M	1.7±1.6	Age: F_3,805_=14.749; p<0.001 Sex: F_1,805_=22.072; p<0.001 Age*sex: F_3,805_=2.656; p=0.047
		F	2.6±2.3	
	3 to 5 years old	M	3.0±1.8	
		F	3.1±1.8	
	6 to 9 years old	M	2.3±1.6	
		F	2.8±1.6	
	10 to 12 years old	M	1.4±1.7	
		F	2.3±1.6	
Playing time with physical activity (hours)	0 to 2 years old	M	1.4±1.5	Age: F_3,805_=18.918; p<=0.001 Sex: F_1,805_=0.543; p=0.461 Age*sex: F_3,805_=0.339; p=0.797
		F	1.2±2.1	
	3 to 5 years old	M	1.3±1.1	
		F	1.3±1.1	
	6 to 9 years old	M	0.7±0.9	
		F	0.7±0.8	
	10 to 12 years old	M	0.6±0.9	
		F	0.6±0.8	
Time of physical activity (hours)	0 to 2 years old	M	0.7±0.9	Age: F_3,805_=2.206; p=0.086 Sex: F_1,805_=0.032; p=0.858 Age*sex: F_3,805_=0.444; p=0.722
		F	0.6±1.4	
	3 to 5 years old	M	0.6±1.0	
		F	0.7±1.0	
	6 to 9 years old	M	0.5±0.8	
		F	0.4±0.6	
	10 to 12 years old	M	0.5±0.7	
		F	0.5±0.8	

SD: standard deviation; ANOVA: analysis of variance; M: male; F: female.

Considering playful screen time, we observed the effect of age and sex, but there is no interaction between age group and sex (Table 1). This playful screen time increases significantly with age (p<0.003), and boys present higher numbers than girls (Figure 2).

For the category playing without PA, there was a significant difference between age groups, sex and interaction between age and sex (Table 1). G2 is the group that is most involved in playing without PA (p<0.001 in comparison to G1 and p=0.002 in relation to G3 and G4). There was a difference between G3 and G4 (p<0.001): G3 plays without PA more than G4 (Figure 2). Regarding gender, there is a difference between boys and girls: girls play without PA more than boys (Figure 2). These differences are significant in G1 (p=0.001) and G4 (p=0.001) (Figure 2).

As to the category playing with PA, the analysis revealed there is no difference between genders, but significant reduction with age (Table 1). This expressive reduction occurs between the two youngest and the two oldest groups (significance values higher than 0.001), however, not between G1 and G2 (p=0.681) nor between G3 and G4 (p=0.234) (Figure 2).

In the PA category, we observed there was little time dedicated to that activity, without differences between age groups and sex (Table 1).

**Figure 3:**
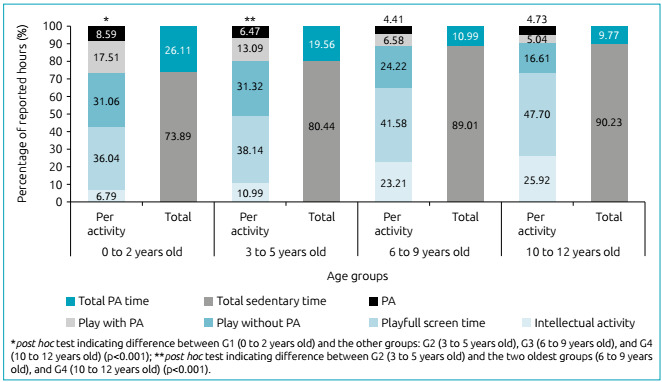
Mean percentage of time in which children spent performing different activities, general physical activity and sedentary time, during the social isolation, as reported by the parents.

To better understand the relative importance of each category of activities in the day of the children in the different age groups, the time spent in each activity was converted in percentage, considering the total time reported by parents for all categories. The general PA time and general sedentary time were calculated (Figure 3).

Figure 3 shows that the mean percentage of intellectual activity and playful screen time increase between age groups, whereas the opposite tends to occur between the other categories. It is possible to notice that playing without PA is prevalent in the two youngest groups. We also highlight that the mean percentage of the sedentary time is high in all age groups.

By grouping the categories in total PA time and total sedentary time, the results indicate reduction in the percentage of total PA time (F_3,798_=37.228; p<0.001) and increase in total sedentary time (F_3,798_=37.228; p<0.001) with age.

## DISCUSSION

This study identified the behavior of Brazilian children aged less than 13 years during the first month of social isolation. Our results suggest that time of PA can be compromised in this situation.

The condition of life of these children leads them to more sedentary behaviors than in days with normal school activities, especially while they develop. Our results show much higher numbers of total sedentary time in relation to studies that assessed this time on school days, showing that more than 60% of the time is spent on sedentary activities.^9^ ^,^ ^10^

Studies point that Brazilian children aged more than three years, regardless of social isolation, have spent around 2.5 hours on screen activity,^11^ which is above the recommendations of the Brazilian Society of Pediatrics (SBP).^12^ This contributes with a sedentary behavior, reducing the opportunity for the children to be physically active, and is related with: parental concern about safety, preventing the kids from performing outdoor activities; high demand of activities related to the parents jobs; unfavorable structural conditions in specific neighborhoods, decreasing the changes of a more active lifestyle; great availability of computer games and TV shows, which encourage sedentary activities.^13^ Our findings indicate that social isolation led to higher playful screen time, therefore leading to increased sedentary time and reduced total PA time, as reported by the parents.

During the school period, the routine of the children is more structured, and can generate healthier behaviors regarding the practice of PA, sleep and diet.^14^ A more structured routine provides opportunities both in school and in extracurricular sports activities, so that the children can practice PA and obtain the recommendation for moderate or vigorous PA. The literature suggests reduction in moderate or vigorous PA and increase in sedentary behavior while children grow up.^14^ ^,^ ^15^ Our results did not demonstrate this unfavorable tendency, probably because the times of PA were very low for all ages. No tendency was found to show that girls are more sedentary than boys,^14^ ^,^ ^15^ possibly because the children are in social isolation due to the pandemic.

Based on the information that some of these children are attending online classes, and, in this sense, there is a variety of strategies adopted by the schools, in the beginning of the isolation period we observed increased screen time not only for studying, but also for leisure purposes, therefore surpassing the daily limits of screen time recommended by the SBP^12^ (for children younger than two years, screen exposure should be prevented, without the need for it; from two to five years of age, one hour a day at most, always with supervision; from six to ten years of age, the limit should be from to two hours a day, with supervision; and after the age of 11, from two to three hours a day).

Screen time can be very much influenced by the use of social media, which is the only way to keep in touch with family and friends during the isolation period, and it is also related to games or watching TV. The increase in this type of sedentary activity may contribute with weight gain for these children^16^ and favor the early onset of chronic diseases.^17^ Studies show that children who watch TV for more than three hours a day have 65% more chances of being obsess when compared to shoe who watch less than hour a day.^18^ Having a computer, videos or game devices in the bedroom also increases the chances of sedentary behavior among the children.^12^ ^,^ ^19^

Intellectual activities are the prevalent type of activity reported by the parents after the age of three years. The percentage of these activities in the routine of the children in comparison to the other reported activities increases with age, and in groups of children aged from six to nine, and ten to 12, the children spend four hours a day, in average, on organized intellectual activities or playful screen activities. Such a result was expected, because children aged from six to 12 years attend Elementary School, and often have many school chores to be executed during the confinement period. Besides the school activities, the time spent on activities involving playing without PA was higher than one hours, regardless of the age group.

After the age of six, the children present reduced playing time, if compared to those aged less than five. Inversely to intellectual activities, playing activities reduce while children grow up. In this sense, until the age of five, the activities organized in school are based on the field of experience, reinforcing the idea that the child learns through concrete, interactive, playful and integrative activities of several fields of knowledge,^20^ which is based on the stages of neuropsychomotor development.^21^ ^,^ ^22^ From the age of six on, the children experience major changes in development, which reflect on their relationship with themselves, others, and the world. The school organization changes and is based on the progression of knowledge, with the consolidation of the previous learning process and the amplification of language practices and the aesthetic and intercultural experience of the children.^20^ The focus on cognitive and social skills is a priority.^23^

Even though social isolation is necessary and efficient to prevent the transmission of SARS-CoV-2,^6^ our results suggest that the strategy is harmful for the levels of PA of the children, as demonstrated in previous studies.^24^ ^,^ ^25^ We verified that the prolonged permanence in the household leads to increasing sedentary behaviors, such as spending too much time sitting or lying down for activities such as playing, watching TV< using mobile devices, besides the reduction of regular PA and involvement in activities that favor the early onset of chronic diseases.^21^ ^,^ ^22^

Staying at home is a fundamental security step that can limit the dissemination of SARS-CoV-2, but it can contribute with anxiety and depression, which, on the other hand, can lead to a sedentary lifestyle and result in a series of chronic health conditions.^25^ It is possible that stress factors, such as prolonged confinement, fear of infection, frustration and boredom, inadequate information, lack of personal contact with classmates, friends and teachers, lack of personal space at home and financial losses in the family, may cause more problems and long-term effects on children and adolescents.^26^ ^,^ ^27^ ^,^ ^28^

The parents reported that family activities increased in this period. Confinement in the household can provide opportunities to improve the interaction between parents and children, to involve the children in family activities and to improve their self-sufficiency skills.^24^ Children are vulnerable to environmental risks. Their physical and mental health, and their behavior throughout life, are deeply rooted in the early years.^29^

Based on these results, there are implications to be considered by the professionals involved in public health, researchers and parents, focusing on the fight against physical inactivity, with the possibility to build preventive strategies against sedentary lifestyles, able to be implemented in the household environment and minimize the impact of this isolation on health.

The consequences of this forced lifestyle resulting from the SARS-CoV-2 pandemic will be experienced much later, after the end of isolation; but a better understanding of these effects will be possible if a complete description of this period is carried out.^30^ We hope to contribute with the characterization of the routines of the social isolation period and be able to create strategies addressed to the specific motivation of each age group, associated with the strategies of the families, in order to reduce the sedentary time among the children. The study will continue throughout the entire isolation period, offering a complete image of the routines in the families.

This study’s limitation is the lack of information about the social and economic status of the families and the region of the country where they live. It provides a first approach on the household routines of Brazilian families and their impact on the time of PA of the children who are living in social isolation. The results point to a strong reduction in the time of PA throughout the childhood period, when children are forced to stay inside their houses. Screen time increased in the age groups, being higher among boys, but there was no difference between genders in general PA.

## References

[1] Guo YR, Cao QD, Hong ZS, Tan YY, Chen SD, Jin HJ (2020). The origin, transmission and clinical therapies on coronavirus
disease 2019 (COVID-19) outbreak - an update on the status. Mil Med Res.

[2] Huang C, Wang Y, Li X, Ren L, Zhao J, Hu Y (2020). Clinical features of patients infected with 2019 novel
coronavirus in Wuhan, China. Lancet.

[3] Sun P, Lu X, Xu C, Sun W, Pan B (2020). Understanding of COVID-19 based on current
evidence. J Med Virol.

[4] Johns Hopkins University & Medicine COVID-19 Dashboard by the Center for Systems Science and Engineering
(CSSE) at Johns Hopkins University (JHU).

[5] Brazil - Ministério da Saúde COVID-19: painel coronavírus. Brasília: Ministério da Saúde.

[6] Marques ES, Moraes CL, Hasselmann MH, Deslandes SF, Reichenheim ME (2020). Violence against women, children, and adolescents during the
COVID-19 pandemic: overview, contributing factors, and mitigating
measures. Cad Saúde Pública.

[7] Brazil - Ministério da Educação Coronavírus (COVID-19).

[8] Hesketh KR, Lakshman R, van Sluijs EM (2017). Barriers and facilitators to young children’s physical activity
and sedentary behaviour: a systematic review and synthesis of qualitative
literature. Obes Rev.

[9] Verloigne M, Ridgers ND, Chinapaw M, Altenburg TM, Bere E, van Lippevelde W (2017). Patterns of objectively measured sedentary time in 10- to
12-year-old Belgian children: an observational study within the ENERGY
project. BMC Pediatr.

[10] Abbott RA, Straker LM, Mathiassen SE (2013). Patterning of children’s sedentary time at and away from
school. Obesity.

[11] Araújo LG, Turi BC, Locci B, Mesquita CA, Fonsati NB, Monteiro HL (2018). Patterns of physical activity and screen time among Brazilian
children. J Phys Act Health.

[12] Sociedade Brasileira de Pediatria (2019). Manual de Orientação. Grupo de Trabalho Saúde na Era Digital
(2019-2021). #Menos telas #mais saúde.

[13] Silva PV, Costa AL (2011). Physical activity effects on the health of children and
adolescents. Psicol Argum.

[14] Fu Y, Brusseau TA, Hannon JC, Burns RD (2017). Effect of a 12-week summer break on school day physical activity
and health-related fitness in low-income children from CSPAP
Schools. J Environ Public Health.

[15] Kann L, McManus T, Harris WA, Shanklin SL, Flint KH, Hawkins J (2018). Youth risk behavior surveillance - United States,
2017. MMWR Surveill Summ.

[16] Morales-Ruán M, Hernández-Prado B, Gómez-Acosta LM, Shamah-Levy T, Cuevas-Nasu L (2009). Obesity, overweight, screen time and physical activity in Mexican
adolescents. Salud Publica Mex.

[17] Santos A, Andaki AC, Amorim PR, Mendes EL (2013). Factors associated with sedentary behavior in school children
aged between 9 and 12. Motriz Rev Educ Fis.

[18] Singh GK, Kogan MD, van Dyck PC, Siahpush M (2008). Racial/ethnic, socioeconomic, and behavioral determinants of
childhood and adolescent obesity in the United States: analyzing independent
and joint associations. Ann Epidemiol.

[19] Tandon P, Grow HM, Couch S, Glanz K, Sallis JF, Frank LD (2014). Physical and social home environment in relation to children’s
overall and home-based physical activity and sedentary time. Prev Med.

[20] Brazil - Ministério da Educação Base Nacional Comum Curricular.

[21] McCoy DC, Black MM, Daelmans B, Dua T Early childhood matters. Measuring development in children from birth to
age 3 at population level.

[22] Adolph KE, Franchak JM (2017). The development of motor behavior. WIREs Cogn Sci.

[23] Carson V, Kuzik N, Hunter S, Wiebe SA, Spence JC, Friedman A (2015). Systematic review of sedentary behavior and cognitive development
in early childhood. Prev Med.

[24] Wang G, Zhang Y, Zhao J, Zhang J, Jiang F (2020). Mitigate the effects of home confinement on children during the
COVID-19 outbreak. Lancet.

[25] Chen P, Mao L, Nassis GP, Harmer P, Ainsworth BE, Li F (2020). Wuhan coronavirus (2019-nCoV): the need to maintain regular
physical activity while taking precautions. J Sport Health Sci.

[26] Brooks SK, Webster RK, Smith LE, Woodland L, Wessely S, Greenberg N (2020). The psychological impact of quarantine and how to reduce it:
rapid review of the evidence. Lancet.

[27] United Nations Educational, Scientific and Cultural
Organization (2020). Covid-19 educational disruption and response.

[28] The Alliance for Child Protection in Humanitarian Action Technical note: protection of children during the coronavirus pandemic
(V.2). Alliance for Child Protection in Humanitarian Action.

[29] Clark H, Coll-Seck AM, Banerjee A, Peterson S, Dalglish SL, Ameratunga S (2020). A future for the world’s children? A WHO-UNICEF-Lancet
Commission. Lancet.

[30] Núcleo Ciência Pela Infância (2020). Comitê Científico Núcleo Ciência pela Infância. Repercussões da pandemia
COVID-19 no desenvolvimento infantil.

